# A Combined Method Based on the FIPV N Monoclonal Antibody Immunofluorescence Assay and RT-nPCR Method for the Rapid Diagnosis of FIP-Suspected Ascites

**DOI:** 10.1155/2023/8429106

**Published:** 2023-03-28

**Authors:** Liang Xu, Shaotang Ye, Yulin Ding, Yuqing Xiao, Congwen Yao, Zhen Wang, Siqi Cai, Jiajun Ou, Jianwei Mao, Xuerui Hu, Song Cheng, Jingyu Wang, Gang Lu, Shoujun Li

**Affiliations:** ^1^College of Veterinary Medicine, South China Agricultural University, Guangzhou, China; ^2^Guangdong Provincial Key Laboratory of Prevention and Control for Severe Clinical Animal Diseases, Guangzhou, China; ^3^Guangdong Technological Engineering Research Center for Pet, Guangzhou, China; ^4^College of Veterinary Medicine, Inner Mongolia Agricultural University/Key Laboratory of Clinical Diagnosis and Treatment Technologyin Animal Disease, Ministry of Agriculture, Hohhot 010018, China; ^5^Laboklin Laboratory for Clinical Diagnostics, Guang Zhou 510700, China

## Abstract

Feline infectious peritonitis (FIP), which is caused by feline infectious peritonitis virus (FIPV), is a fatal and immunologically mediated infectious disease among cats. At present, due to the atypical clinical symptoms and clinicopathological changes, the clinical diagnosis of FIP is still difficult. The gold standard method for the differential diagnosis of FIP is immunohistochemistry (IHC) which is time-consuming and requires specialized personnel and equipment. Therefore, a rapid and accurate clinical diagnostic method for FIPV infection is still urgently needed. In this study, based on the etiological investigation of FIPV in parts of southern China, we attempted to explore a new rapid and highly sensitive method for clinical diagnosis. The results of the etiological investigation showed that the N gene of the FIPV BS8 strain had the highest homology with other strains. Based on this, a specific FIPV BS8 N protein monoclonal antibody was successfully prepared by expression of the recombinant proteins, immunization of mice, fusion and selection of hybridoma cell lines, and screening and purification of monoclonal antibodies. Furthermore, we carried out a time-saving combination method including indirect immunofluorescence assay (IFA) and nested reverse transcription polymerase chain reaction (RT-nPCR) to examine FIP-suspected clinical samples. These results were 100% consistent with IHC. The results revealed that the combined method could be a rapid and accurate application in the diagnosis of suspected FIPV infection within 24 hours. In conclusion, the combination of IFA and RT-nPCR was shown to be a fast and reliable method for clinical FIPV diagnosis. This study will provide insight into the exploitation of FIPV N antibodies for the clinical diagnosis of FIP-suspected ascites samples.

## 1. Introduction

FIP is one of the most important infectious diseases in cats and is caused by infection with feline coronavirus (FCoV). FIPV is a nonsegmented, positive-sense, enveloped, and single-stranded RNA virus that belongs to the family Coronaviridae [1, 2]. FIPV has 11 putative open reading frames (ORFs), including polyproteins with RNA synthesis functions, four structural proteins (highly glycosylated spike proteins (S), envelope proteins (E), membrane proteins (M), nucleocapsid proteins (N)), and several nonstructurally accessory proteins [3–5]. FCoV is divided into type I and type II based on its serological characteristics [4, 6]. FCoV type II arises after recombination between FCoV type I and canine coronavirus (CCV) [7, 8]. Both FCoV type I and type II can cause FIP [8].

It has been reported that mainly young cats aged 6 months to 2 years and male cats are affected by FIPV [9, 10]. It has been documented that approximately 0.3% to 1.4% of feline deaths are caused by FIP [11]. FIPV can efficiently and continuously replicate in monocytes and macrophages [12]. Monocyte/macrophage-tropic FIPV typically manifests as either effusive (wet form) or noneffusion (dry form) [13, 14]. The wet form is characterized by immune-mediated, fibrinous-granulomatous serositis, often with protein-rich effusions in the thoracic or abdominal cavities [15]. The dry form of FIP generally lacks cavitary effusion and is instead characterized by pyogranulomatous lesions formation in a variety of body organs and around blood vessels [16]. In many cases, the clinical symptoms of FIP (chronic fever, weight loss, anorexia, and malaise) are atypical, and the clinicopathological changes are not specific to FIP, such as lymphocytopenia, neutrophilia, hyperproteinemia, and hyperglobulinemia [17].

FIPV infection in cats is fatal without proper and specific treatment. The diagnosis of FIP is challenging owing to the lack of pathognomonic clinical signs or laboratory changes, especially when no effusion is present [17, 18]. Therefore, the ability to perform a precise and rapid diagnosis for FIP is critical. To date, several methods have been applied in the clinical diagnosis of FIP. The Rivalta's test is simple and inexpensive and has been used to differentiate transudates from exudates [19]. This test has been frequently used in feline effusions, but it occasionally produces false-positive results [20]. According to the research results of Yvonne Fischer, 5.7% of the exudates with positive results of the Rivalta's test were classified as transudate, and only 58.6% of the exudates with positive results of the Rivalta's test were FIP, and 41.4% of the cats with positive results of the Rivalta's test had other diseases [21]. Since the first application of RT‒PCR in FCoV detection, it has been used to amplify FCoV RNA in different materials for FIP diagnosis [22, 23]. However, FCoV RNA can also be detected outside of the gastrointestinal tract in cats without FIP infection [24]. A reverse transcription-recombinase polymerase amplification (RT-RPA) method was used for rapid FCoV detection [25]. RT-RPA has been used to detect the nucleic acid sequences from a wide variety of pathogens within a 15-min assay run-time [25, 26]. The rapid results were achieved using additional proteins to separate the DNA strands instead of thermal cycling as in the PCR [25]. RT-PRA is also a method for screening cat populations for FCoV infections. Nevertheless, it is not designed specifically for FIPV infection [25]. The detection of antibody production against FCoV is specific for infection [27–29]. However, high antibody titers can also be detected in the blood of feline enteric coronavirus-infected (FECV) cats, which is still not a specific indicator for FIP [30]. In addition, gene detection has been reported to be an efficient method for viral detection. RT-nPCR has previously been reported to detect FCoV and compared with RT‒PCR, this method can effectively detect asymptomatic infected cats [31]. The sensitivity and specificity of RT-nPCR for detecting FIPV in ascites or exudate were 100% [17, 32, 33]. However, it requires auxiliary validation by other methods.

As a specific biological material, monoclonal antibodies have become key components in various clinical laboratory diagnostic tests. Their widespread application in detecting and identifying serum analytes, cell markers, and pathogenic agents has largely arisen through the exquisite specificity of these unique reagents [34, 35]. Monoclonal antibodies recognize only a single epitope [36]. In this study, the monoclonal antibody prepared was used to detect FIPV antigen by IFA and IHC. RT-nPCR was used to detect FIPV 3′ untranslated region (3′UTR) genes, N genes, and S genes. IFA combined with RT-nPCR improved the detection accuracy of FIPV.

The aim of this study was to establish a rapid and accurate method for the detection of FIPV. A systematic FIPV detection method combining IFA with RT-nPCR was initially established, which provided a new idea for exploiting FIPV antibodies and diagnosing FIP.

## 2. Materials and Methods

### 2.1. Cells, Primary Reagents, Vectors, and Experimental Animals

*Felis catus* whole fetus 4 (Fcwf-4) cells were purchased from an American-type culture collection (ATCC, USA). Crandell-Rees feline kidney (CrFK) cells and human embryonic kidney (HEK) 293T cells were stored in our laboratory. All cells were cultured at 37°C and 5% (v/v) CO_2_ in Dulbecco's modified Eagle medium (DMEM; Biological Industries, Kibbutz Beit-Haemek, Israel) with 10% fetal bovine serum (FBS) (Biological Industries). Mouse myeloma cells (SP2/0) were purchased from GeneCreate (GeneCreate, Wuhan, China) and were cultured in RPMI-1640 medium (Invitrogen, Carlsbad, CA) supplemented with 10% FBS. Reverse transcription Kit HiScript III 1st Strand cDNA Synthesis Kit (+gDNA Wiper), Phanta Max Super-Fidelity DNA Polymerase, and FastPure Gel DNA Extraction Mini Kit were purchased from Vazyme (Vazyme, Nanjing, China). A SimplyP animal pathogen DNA/RNA coextraction kit was purchased from BioER (BioER, China). Factor Xa Protease and amylose resins were purchased from NEB (NEB, USA). The diluent of the primary immunostaining antibody and secondary immunostaining antibody and DAPI were purchased from Beyotime (Beyotime, Shanghai, China). The pMAL-C5X vector and pCMV-MYC vector were purchased from GeneCreate (GeneCreate, Wuhan, China). Alexa Fluor 488-conjugated fluorescent secondary antibody was purchased from ABclonal (ABclonal, Wuhan, China). BALB/c mice were purchased from Hubei Provincial Laboratory Animal Public Service Center (Wuhan, China).

### 2.2. Sample Collection

In this study, 152 ascites samples whose Rivalta's tests were positive were collected from animal hospitals in Guangzhou and Zhejiang from 2018 to 2021, of which samples collected from Guangzhou (*n* = 117) and samples collected from Zhejiang (*n* = 35) were all stored at −80°C.

### 2.3. Detection of FIPV by RT-nPCR

The 3′UTR gene of FIPV was detected by RT-nPCR as described in a previous study [32], and the FIPV S gene was amplified as described by Addie et al. The N gene of FIPV was detected as described in a previous study [37]. FIPV RNA was extracted from 152 ascites samples using the SimplyP animal pathogen DNA/RNA coextraction kit. RNA extraction was carried out according to the manufacturer's instructions. A Hiscript III 1st Strand cDNA Synthesis Kit was used to synthesize FIPV cDNA. The reverse transcription system was as follows: 8 *µ*L of extracted RNA premix, 65°C for 5 min, ice for 2 min, 2 *µ*L of 5× gDNA wiper mix, mix by pipette gently, 42°C for 2 min, add 2 *µ*L of HiScript III Enzyme Mix, 2 *µ*L of 10× RT Mix, 1 *µ*L of random hexamers, and RNase-free ddH_2_O supplemented to a total volume of 20 *µ*L. The cDNA synthesis reaction conditions were as follows: 25°C for 5 min, 50°C for 45 min, 85°C for 2 min, and the product was stored at −20°C. PCR amplification was performed according to Phanta Max Super-Fidelity DNA Polymerase. The reaction system was prepared as follows: 25 *µ*L of 2× Phanta Max Buffer, 1 *µ*L of dNTPs, 2 *µ*L of forward primer, 2 *µ*L of reverse primer, 1 *µ*L of Phanta Max Super-Fidelity DNA Polymerase, 5 *µ*L of cDNA, and RNase-free ddH_2_O supplemented to a total volume of 50 *µ*L. The amplification procedure was set as follows: predenaturation at 95°C for 3 min, denaturation at 95°C for 15 sec, annealing at 54°C for 15 sec, extension at 72°C for 2 min, amplification for 35 cycles, and a final extension at 72°C for 5 min.

### 2.4. Phylogenetic Analysis of the FIPV N Gene and S Gene

RT-nPCR products were purified and sequenced by the Sanger method (Sangon Biotech, China). Mega X software (Auckland, New Zealand) was used to construct genetic evolutionary trees for the N and S genes. The neighbor-joining phylogenetic tree was constructed using the *p*-distance model and 1,000 bootstrap replications. Amino acid and nucleotide similarity were performed using MegaAlign 8.1 software (DNASTAR Inc., WI, USA).

### 2.5. Preparation of Monoclonal Antibody against FIPV N Protein

According to the etiological investigation and analysis, the strain with the highest amino acid homology of the *N* gene was selected. The monoclonal antibody preparation was carried out according to the protocol described previously [34, 38]. Briefly, the recombinant BS8 nucleocapsid protein and Freund's incomplete adjuvant were mixed and injected subcutaneously into SPF BALB/C mice. The immune process of mice was commissioned by GeneCreate (GeneCreate, Wuhan, China). The splenic lymphocytes from the immunized mice were fused with SP2/0 myeloma cells at of 10 : 1 using a polyethylene glycol (PEG) reagent. The fused cells were first cultured in a Hypoxanthine-Aminopterin-Thymidine (HAT) medium for 7 days. The positive cell lines were selected and the cell supernatant was used for IFA. The subtypes of antibodies were identified by combining 6 subtypes of secondary antibodies using ELISA. The selected hybridoma cells were seeded into the abdominal cavity of mice a week later. After 7–10 days, ascites were collected and purified by a Protein G-agarose affinity chromatography column, and the purified monoclonal antibodies were verified by SDS‒PAGE and IFA.

### 2.6. The Application of the IFA

To better detect FIPV, the FIPV BS8 N monoclonal antibody was used to detect infected FIPV samples. To detect FIPV-infected Fcwf-4 cells, the cells were washed three times with PBS and fixed with 4% paraformaldehyde for 10 min. The fixed cells were blocked with QuickBlockTM blocking solution and incubated for 15 min. The blocked FCWF-4 cells were incubated with FIPV-BS8 N monoclonal antibody overnight at 4°C. Cells were washed with PBST (PBS containing 0.1% Tween-20) for 3 min each. Then, the cells were incubated with Alexa Fluor® 488 goat antimouse IgG secondary antibody for 1 hour at room temperature in the dark. After washing three times with PBST for 3 min each, the cells were incubated with DAPI at room temperature for 5 min and washed with PBST three times for 5 min each. Finally, the cells were observed with a laser confocal microscope.

For the ascites samples, the ascites samples were centrifuged at 1000 rpm/min for 5 min, and the precipitate and a small amount of supernatant were absorbed and transferred to a sterile centrifuge tube, which was thoroughly mixed. A drop was taken on the slide, and the slide was pushed by the cross-section method and left to dry at room temperature. The above-given steps were followed, and the cells were observed under a confocal laser microscope. In this experiment, 36 ascites samples were randomly selected for IFA and RT-nPCR detection.

### 2.7. Immunohistochemistry

Postmortem autopsy of FIP-infected cats was performed on small intestinal tissue for immunohistochemistry (IHC). IHC procedures were carried out according to the standard protocol [39].

## 3. Results

### 3.1. Sample Statistics and Detection of FIPV

A total of 152 suspected FIPV-positive ascites samples were collected from animal hospitals in Guangdong and Zhejiang, including 117 from Guangzhou and 35 from Hangzhou. The FIPV 3′UTR, N, and S genes were detected by RT-nPCR amplification. The agarose gel electrophoresis results of some samples are listed in Supplemental Figure S1. Positive samples were detected in 96 out of 152 samples (63.16%, 96/152). Seventy-nine strains of the FIPV partial S gene were successfully amplified by RT-nPCR. Sixty-five FIPV partial S gene sequences were successfully obtained, including 64 FIPV type I strains (98.46%, 64/65) and 1 FIPV type II strain (1.54%, 1/65). Information on the successfully sequenced N and S gene samples is listed in Supplementary Table S1.

### 3.2. Phylogenetic Analysis of the S Gene and N Gene

**Figure 1:**
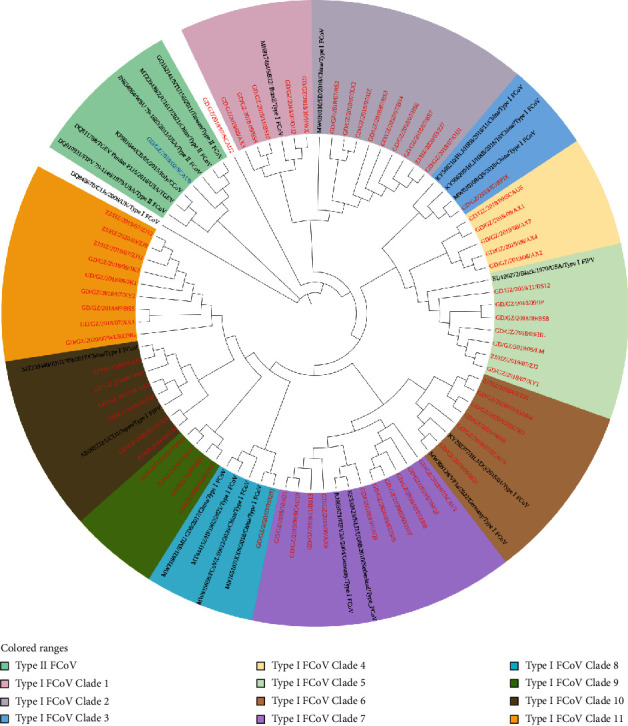
Phylogenetic analysis of the FIPV partial S gene (360 bp). The reference sequences downloaded from GenBank are marked in black. The FIPV type I strains marked in red were 64, and the FIPV type II strain marked in blue was the SCAU9 strain. Neighbor-joining trees were constructed with 1,000 bootstrap replicates.

A total of 65 FIPV partial S gene sequences could be divided into two groups, type I and type II. The FIPV S gene phylogenetic tree showed that the type I obtained could be divided into 11 evolutionary clades. Only the SCAU9 strain was FIPV type II (Figure 1). FIPV N gene phylogenetic analysis showed that the strains were relatively dispersed. The FIPV N gene phylogenetic tree is listed in Supplemental Information Figure S2. The nucleotide and amino acid homology of FIPV S and N genes are listed in Supplemental Information Figures S3 and S4.

### 3.3. Preparation of Monoclonal Antibody against FIPV BS8 N

**Figure 2:**
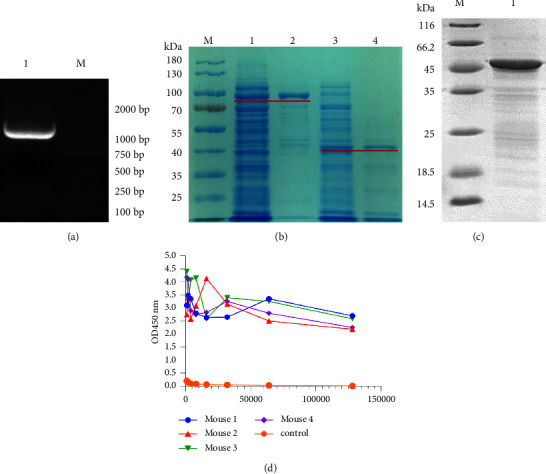
(a) Identification of the amplified products of the FIPV BS8 N gene. Lane 1, FIPV BS8 N, lane M DL2000 marker. (b) Identification of the expressed and purified products of FIPV BS8 N recombination protein by SDS‒PAGE, lane M, protein marker; lane 1, pMAL-C5X–BS8-N with IPTC; lane 2, pMAL-C5X–BS8-N expression product purified by affinity chromatography; lane 3, purified pMAL-C5X–BS8-N protein cleaved by Xa Factor; lane 4, GE HiTrap Q FF purified pMAL-C5X–BS8-N protein without MBP. (c) Identification of the expressed and purified products of the FIPV BS8 N recombination protein by Western blotting. Lane M protein marker; lane 1, purified pMAL-C5X–BS8-N protein. (d) Mouse serum titers after booster immunization. Serially diluted serum samples were tested using an indirect ELISA, and serum samples from unimmunized mice served as the negative control.

The amino acid identity of the N gene of the BS8 strain was more than 91% with that of other strains obtained in this study. Therefore, the BS8 strain N gene was selected as the template for the preparation of the antibody. The N gene of the BS8 strain (1134 bp) (Figure 2(a)) was amplified and cloned into a pMAL-C5X. The recombinant protein size was approximately 100 kDa due to the presence of the MBP tag (Figure 2(b) Lane 1). The FIPV BS8 N protein was approximately 42 kDa in size after the removal of the MBP tag (Figure 2(b), lane 4). Western blot was used to analyze the protein purity of the pMAL-C5X–BS8-N protein after purification (Figure 2(c)). Antiserum titers of immunized mice were assessed by ELISA. After four booster immunizations, immunized mice showed high antibody titers (1 : 32,000–1 : 128,000) compared with nonimmunized mice (Figure 2(d)).

**Figure 3:**
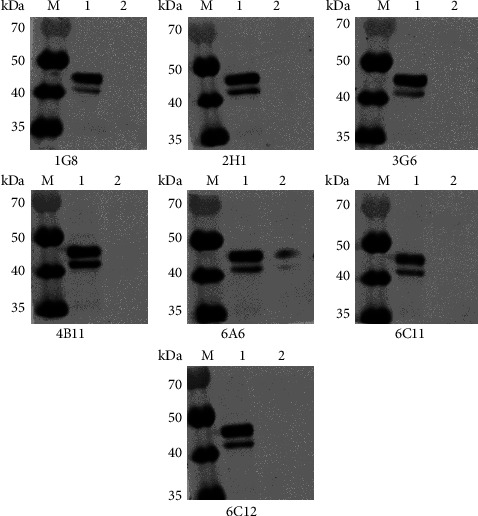
Screening of antibodies by Western blot. CrFK cells were transfected with the pCMV-MYC-BS8-N. Protein was extracted from CrFK cells at 24 h post-transfection. Lane M, protein marker, lane 1, pCMV-MYC-BS8-N, lane 2, pCMV-MYC vector.

After cell fusion, 30 positive cell lines were selected for monoclonal cell screening. The supernatants of positive cell lines were collected as primary antibodies for IFA detection. The IFA results are listed in Supplement Informational Figures S5–S7. The IFA results showed that the supernatant antibodies of cell lines 1G8, 2H1, 2F5, 4B11, 4B12, 4E7, 5E2, 5F11, 6A6, 6C8, and 6F12 showed strong fluorescence signals. After transfection of CrFK cells with the pCMV-MYC-BS8-N, the monoclonal cell lines were screened by western blot. Western blot results showed significant bands in the supernatants of the 1G8, 2H1, 3G6, 4B11, 6A6, 6C11, and 6C12 cell lines (Figure 3). A total of three subtypes of monoclonal antibodies were obtained by ELISA: 15 strains of IgG1 type, 1 strain of IgG2a type, and 2 strains of IgG2b type. According to the above results, the 4B11 cell line with the best performance was finally selected for antibody purification.

**Figure 4:**
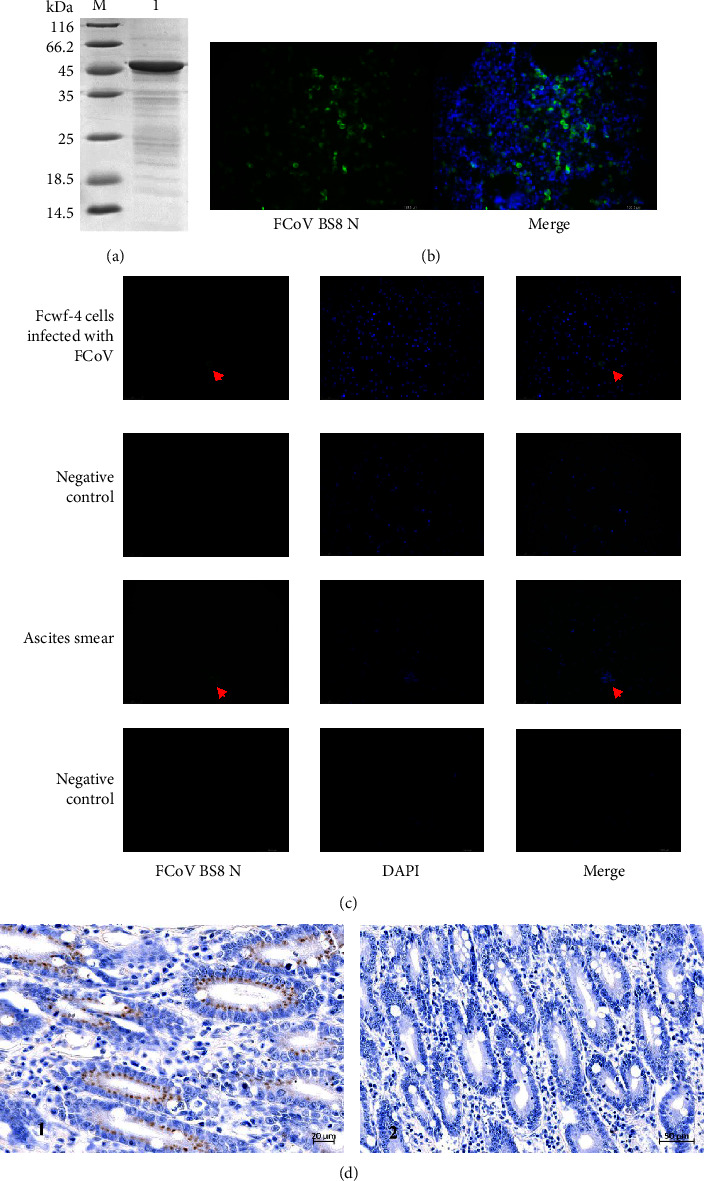
(a) Purification of monoclonal antibody 4B11 by SDS‒PAGE. Lane M, protein marker, lane 1, the purified protein BS8 N. (b) Identification of purified 4B11 antibody by IFA. HEK 293T cells were transfected with the pCMV-MYC-BS8-N. The purified antibody 4B11 was used as the primary antibody for IFA. Scale bar = 75 *μ*m. (c) The detection of FCoV by IFA. FCoV-infected FCWF-4 cells and ascites with infected macrophagocytes were examined by IFA. Samples without infected FIPV were used as a negative control. Red arrows show infected cells. Green fluorescence represents ALexa Fluor® 488 goat antirabbit IgG secondary antibody. Blue fluorescence indicates nuclei stained with DAPI. Scale bar = 100 *μ*m. (d) IHC of the small intestinal epithelium infected with FIP. (1) The small intestine of cats infected with FIPV revealed a positive FIPV antigen reaction in the cytoplasm of small intestinal epithelial cells. (2) Negative control of IHC. The primary antibody used for IHC was an FIPV BS8 N monoclonal antibody. Scale bar = 20 *μ*m.

The ascites were purified by a Protein G-agarose affinity chromatography column, and the purity of the antibody was above 90% after SDS‒PAGE (Figure 4(a)). In addition, after transfection of HEK 293T cells with the pCMV-MYC-BS8-N, the purified antibody was used as the primary antibody for IFA, and a strong fluorescence signal could be observed under a fluorescence microscope (Figure 4(b)).

### 3.4. The Application of the Monoclonal Antibody

A mouse-derived FIPV BS8-N monoclonal antibody was prepared using the N protein sequence of the FIPV BS8 strain obtained in this study. FCWF-4 cells were infected with FIPV and isolated from ascites samples for 24 h. IFA was performed on FCWF-4 cells infected with FIPV. Fluorescence-labeled FIPV N was observed under a fluorescence microscope (Figure 4(c)). At this time, the FIPV N protein was distributed in the cytoplasm of FCWF-4 cells and existed around the nucleus, which was consistent with the results of Yi-Chen Luo et al.

The ascites samples containing FIPV were centrifuged to enrich the macrophages collected in the ascites. FIPV N protein stained with green fluorescence in the cytoplasm of macrophages could be observed under a fluorescence microscope by IFA. Macrophages labeled with green fluorescence could not be observed in the negative control (Figure 4(c)). The results of IFA and RT-nPCR from 36 ascites samples showed that 33 samples were positive by IFA, and 33 samples were positively detected by RT-nPCR (Table 1). Of the 33 samples, 31 had the 3′UTR gene, 23 had the N gene, and 27 had the S gene (Table 2). In this experiment, three ascites push slides were made for each sample, and the sample was considered positive when FIPV-infected macrophages were observed in all slides by IFA. At least one positive detection was made for the 3′UTR gene, N gene, and S gene, and the sample was considered positive.

According to the above results, IFA can directly detect FIPV in fresh ascites samples, and the ascites sediment containing macrophages is evenly coated in the slides through the cross method for IFA. The presence of FIPV antigen in ascites can be directly detected. The FIPV BS8 N antibody could specifically recognize the FIPV N antigen in macrophages. IHC was carried out to detect the FIPV antigen in tissues by using the FIPV BS8 N monoclonal antibody. IHC indicated that the cytoplasmic antigen reaction of small intestinal epithelial cells was positive (Figure 4(d)1). No antigenic signals were found in the cytoplasm of small intestinal epithelial cells in the control group (Figure 4(d)2). These results indicated that the FIPV BS8 N monoclonal antibody was suitable for detecting FIPV in tissues. Using the IFA method combined with RT-nPCR can improve the accuracy of FIPV detection. The method using the FIPV BS8 N monoclonal antibody provides a new idea for the rapid and accurate detection of FIPV.

## 4. Discussion

It is well known that the clinical diagnosis of FIP is frequently difficult [17, 40]. This is due to its varying symptoms at different stages and in different forms [2]. The production of ascites in cats is often secondary to the development of other diseases. There are many factors that affect the appearance of ascites in cats. Abdominal organ failure, trauma of organ tissues, right-sided heart failure, and other factors may lead to the production of ascites. Therefore, when the cat has ascites, it couldn't be easily determined as FIP, and it is necessary to carry out the Rivalta to preliminarily confirm the property of ascites. FIP is characterized by widespread immune-mediated vasculitis and/or pyogranulomas, manifesting as an effusive form, with high protein effusions in body cavities, or a noneffusive form [41]. According to previous research, approximately 40% of domestic cats are infected with FCoV, while the rate increases to 90% in multicat households [24].

In this study, RT-nPCR was used to detect the FIPV 3′UTR and N and S genes in ascites samples suspected of FIPV infection in southern China. A total of 96 samples (63.16%, 96/152) were positive, which was slightly higher than the results of HEhao Ouyang et al. [5]. Differences in data may be related to the number of samples, the way the samples were collected or stored and the method of detection were adopted. In this study, the collected Rivalta-positive ascites samples were tested by IFA combined with RT-nPCR for FIP detection. Differences in these samples and detection methods may lead to different FIP-positive rates. The prevalence of FCoV type I infection in Europe and the United States is 80%–95%, and the prevalence of type II FCoV is less reported, while the prevalence of FCoV type II in most Asian countries is up to 25% [4]. Seventy-nine N gene and 65 S gene partial sequences were obtained by sequencing. Among the 65 S gene partial sequences, 64 strains (98.46%) were FCoV type I, which was higher than the prevalence of FCoV type I in some European countries and the United States. Only 1 strain (1.54%) was FCoV type II. Most cases of FCoV in Guangzhou are type I, while the prevalence of FCoV type II is low. We speculate that there may be several reasons for the higher prevalence of type I FCoV than type II FCoV. Type I FCoV causes a chronic intestinal infection that can be detected in the intestines of most cats and may last for at least 7 months. During this period, cats are constantly shedding the virus in their feces, increasing the risk of infection in other cats. Type II FCoV was caused by homologous RNA recombination events between type I FCoV and CCV. The reorganization of this template switch occurs randomly. For recombinant viruses to emerge and be established, they need to be not only feasible but also selective. Therefore, most of the natural infections are type I FCoV, and the prevalence of type II FCoV is low.

According to amino acid homology analysis, the amino acid identity of the N gene of the BS8 strain was more than 91% of that of other strains. Therefore, the amino acid sequence of the BS8 N gene was selected as the template for the preparation of monoclonal antibodies. A monoclonal antibody against the FIPV N protein was successfully prepared, and the application of the FIPV BS8 N antibody was further explored, which showed better specificity against domestic strains.

For the application of antibodies, we observed the fluorescence of macrophages infected with FIPV in ascites by IFA. We selected a decision strategy to make three push slides for each Rivalta-positive ascites sample, and if each IFA showed positive results, the sample was determined to be infected with FIPV. RT-nPCR was used to detect the 3′UTR, N, and S genes of FIPV. Of the 36 Rivalta-positive ascites samples, 33 samples were positive by IFA, and 33 samples were positively detected by RT-nPCR. The number of positive samples for each detection method is calculated. The detection rate of IFA is slightly higher. The simultaneous detection of the FCoV 3′UTR gene, S gene, and N gene by RT-nPCR greatly improves the detection efficiency of FCoV. The combination of IFA and RT-nPCR methods provides more accurate detection results. The results were consistent with those of the IHC.

Several methods have been used for the diagnosis of FIP. The first assay to detect anti-FCoV antibodies was developed, and it is now well accepted that the detection of anti-FCoV antibodies in the blood is not a specific indicator for FIP and that the detection of FIPV antibodies in effusions is affected by poor specificity and sensitivity [42]. Detecting an FIPV antigen within an effusion macrophage can be a good approach for viral antigen detection, and those involved mostly choose to target the nucleocapsid (N) protein due to its high-yield production in infected cells [2]. IHC is considered the “gold standard” for FIP diagnosis [5, 13, 43]. However, IHC is a time-consuming and complex procedure that requires specialized personnel and equipment [44, 45]. And, the “gold standard” tends to be regarded as the postmortem demonstration of FIPV in tissues. In the IHC, the choice of paraffin section and frozen section will have a certain impact on the antigenicity of tissue. The high-temperature baking of the paraffin section will destroy the antigenicity of the tissue, thus affecting the test results, while the frozen section is easy to form ice crystals in the cells, destroy the cell structure and lead to the diffusion of antigen. Insufficient antigen retrieval will lead to tissue loss of antigenicity, which will affect the antigen detection rate of IHC. Previously published studies investigating the diagnostic accuracy of the direct immunofluorescence assay (DIF) test in clinical specimens have reported 100% specificity and 57%–95% sensitivity [41]. Gene detection has been reported to be an efficient method for viral detection. The sensitivity and specificity of RT-nPCR for detecting FIPV in ascites or exudate were 100% [17, 32, 33].

**Figure 5:**
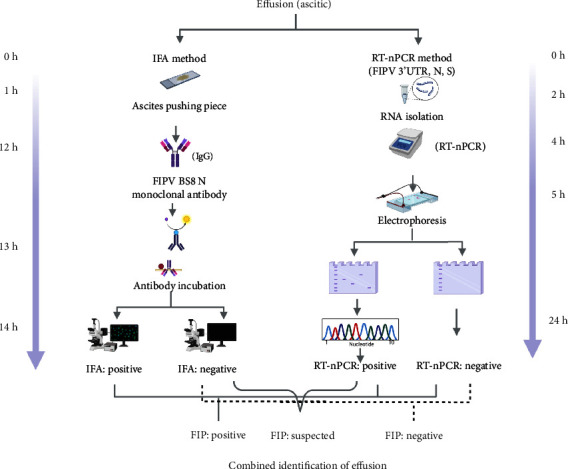
A flowchart of clinical samples through the combined identification of IFA and RT-nPCR. The flowchart is adapted from the “flow chart” by https://BioReender.com (2022). Retrieved from https://app.biorender.com/biorender-templates.

Therefore, the IFA combined with RT-nPCR can improve the accuracy of FIP, and both tests can be easily conducted antemortem. The combined detection method only needs to detect the cat ascites sample, which can reduce the damage to the cat itself to a certain extent and facilitate the timely development of treatment plans. Although both IFA and IHC detect antigens in the sample, the IFA is relatively simple compared to the IHC procedures and could save the detection time. The monoclonal antibody was prepared based on the results of local FIPV etiological investigation results, which would be more suitable for the detection of local FIPV. This combined method detects FIPV simultaneously at the antigen and nucleic acid levels (Figure 5) and could obtain accurate test results within 24 h. And, the diagnostic results are consistent with IHC. The use of the specific monoclonal antibody in this study may provide a new direction for the detection of FIP and lay a foundation for the rapid and accurate diagnosis of FIP.

## 5. Conclusion

FIP is one of the most important infectious diseases in cats. Most cases of FIPV in Guangzhou are FCoV type I, while the prevalence of FCoV type II is low. Based on the etiological investigation of the FIPV N gene, the FIPV N monoclonal antibody was prepared and can be used for the rapid diagnosis of FIP. A systematic FIPV detection method combining IFA with RT-nPCR was initially established, which provided a new idea for exploiting FIPV antibodies and diagnosing FIP.

## Figures and Tables

**Table 1:** The results of ascites samples by IFA and RT-nPCR.

Sample names	IFA	RT-nPCR FCoV 3′UTR gene	RT-nPCR FCoV S gene	RT-nPCR FCoV N gene
1	+	+	+	+
2	+	+	+	+
3	+	+	+	+
4	+	+	+	−
5	+	+	−	+
6	+	+	+	+
7	+	+	−	+
8	+	−	+	+
9	+	+	+	+
10	+	+	+	+
11	−	−	−	−
12	+	−	+	+
13	+	+	−	+
14	+	+	−	−
15	+	+	+	+
16	+	+	+	−
17	+	+	−	+
18	+	+	+	−
19	+	+	−	+
20	+	−	−	−
21	+	+	+	+
22	+	+	−	−
23	+	+	−	+
24	+	+	+	+
25	+	+	−	−
26	+	+	+	+
27	+	+	+	+
28	+	+	+	+
29	+	+	+	+
30	−	+	+	+
31	+	+	−	+
32	+	+	+	+
33	+	+	+	+
34	+	+	+	+
35	+	+	+	+
36	−	−	−	−

Note. “+”: represents positive; “−”: represents negative.

**Table 2:** The number of positive results of ascites samples by IFA and RT-nPCR.

Detection methods	IFA	RT-nPCR FCoV 3′UTR gene	RT-nPCR FCoV S gene	RT-nPCR FCoV N gene
Positive	33	31	23	27

## Data Availability

The data that support the findings of this study are available from the corresponding author upon reasonable request.

## Supplementary Materials

Supplementary Materials Supplemental Figure S1: electrophoresis results of FIPV 3UTR, N, and S genes in some samples. Supplemental Figure S2: genetic evolutionary tree of FIPV N genes. Supplemental Figure S3: the nucleotide homology of S genes of FIPV type I strains with reference strains. Supplemental Figure S4: the nucleotide and amino acid homology of FIPV N genes. Supplemental Figure S5–S7: screening of antibodies by IFA. Supplemental Table S1: the information of the samples obtained sequences of N and S gene of FIPV.
