# Supplementary material for: A Combined Method Based on the FIPV N Monoclonal Antibody Immunofluorescence Assay and RT-nPCR Method for the Rapid Diagnosis of FIP-Suspected Ascites
Source: Transbound Emerg Dis. 2023 Mar 28;2023:8429106. doi: 10.1155/2023/8429106 (PMC12017028; doi:10.1155/2023/8429106)
Supplement: Supplementary Materials — Supplemental Figure S1: electrophoresis results of FIPV 3UTR, N, and S genes in some samples. Supplemental Figure S2: genetic evolutionary tree of FIPV N genes. Supplemental Figure S3: the nucleotide homology of S genes of FIPV type I strains with reference strains. Supplemental Figure S4: the nucleotide and amino acid homology of FIPV N genes. Supplemental Figure S5–S7: screening of antibodies by IFA. Supplemental Table S1: the information of the samples obtained sequences of N and S gene of FIPV. [file 8429106.f1.zip › Supplemental Figures.docx]

FIGURE S1 Electrophoresis results of FIPV 3UTR, N and S genes in some samples. A, B, C and D represent different ascites samples. Lane M, DL 2000, lane 1, the second round of RT-nPCR of FIPV 3’UTR gene, lane 2, the first round of RT-nPCR of FCoV of 3’UTR gene; lane 3, the second round of FIPV N gene; lane 4 the S gene of FIPV type I; lane 5, the S gene of FIPV type II.


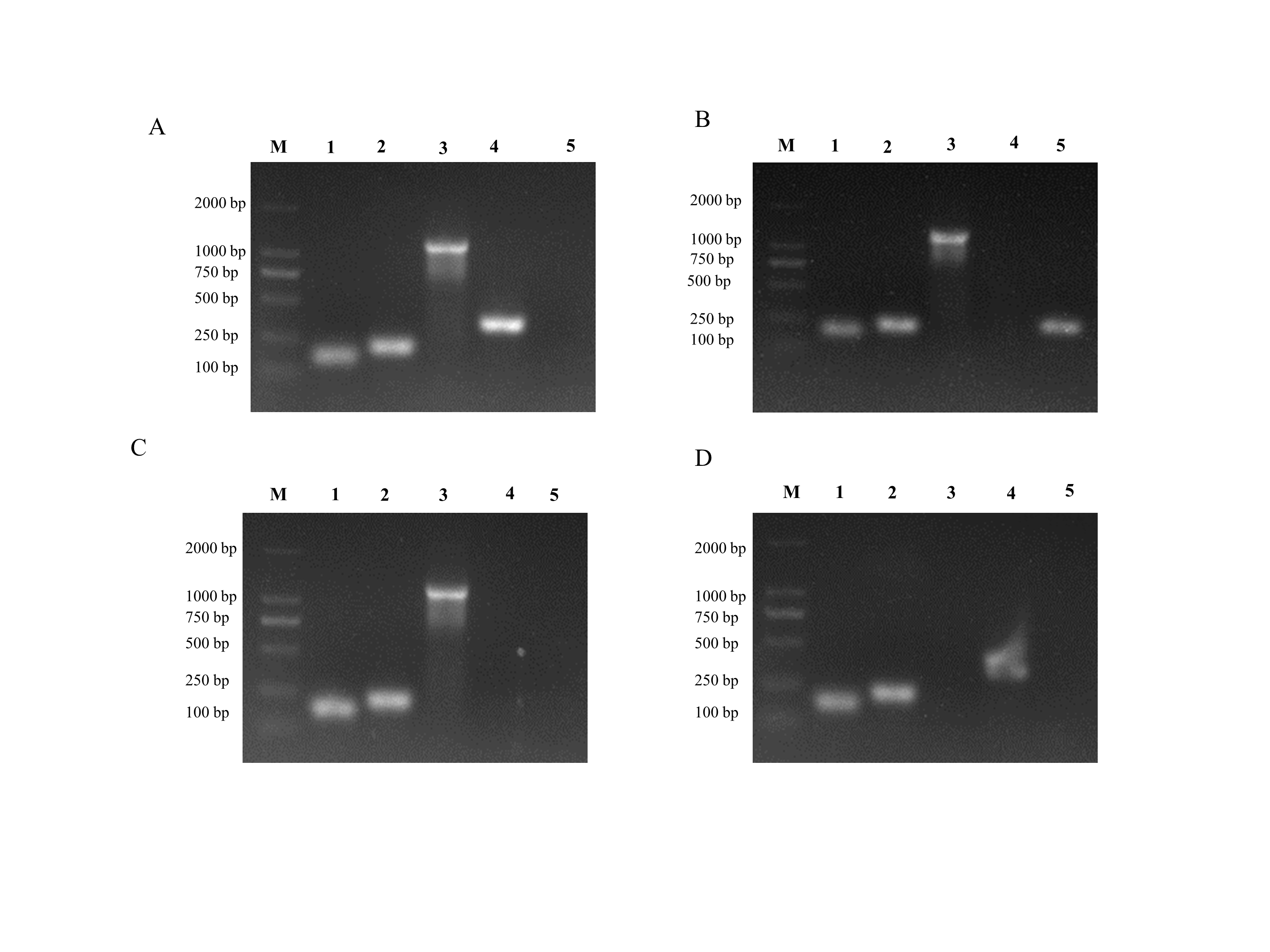


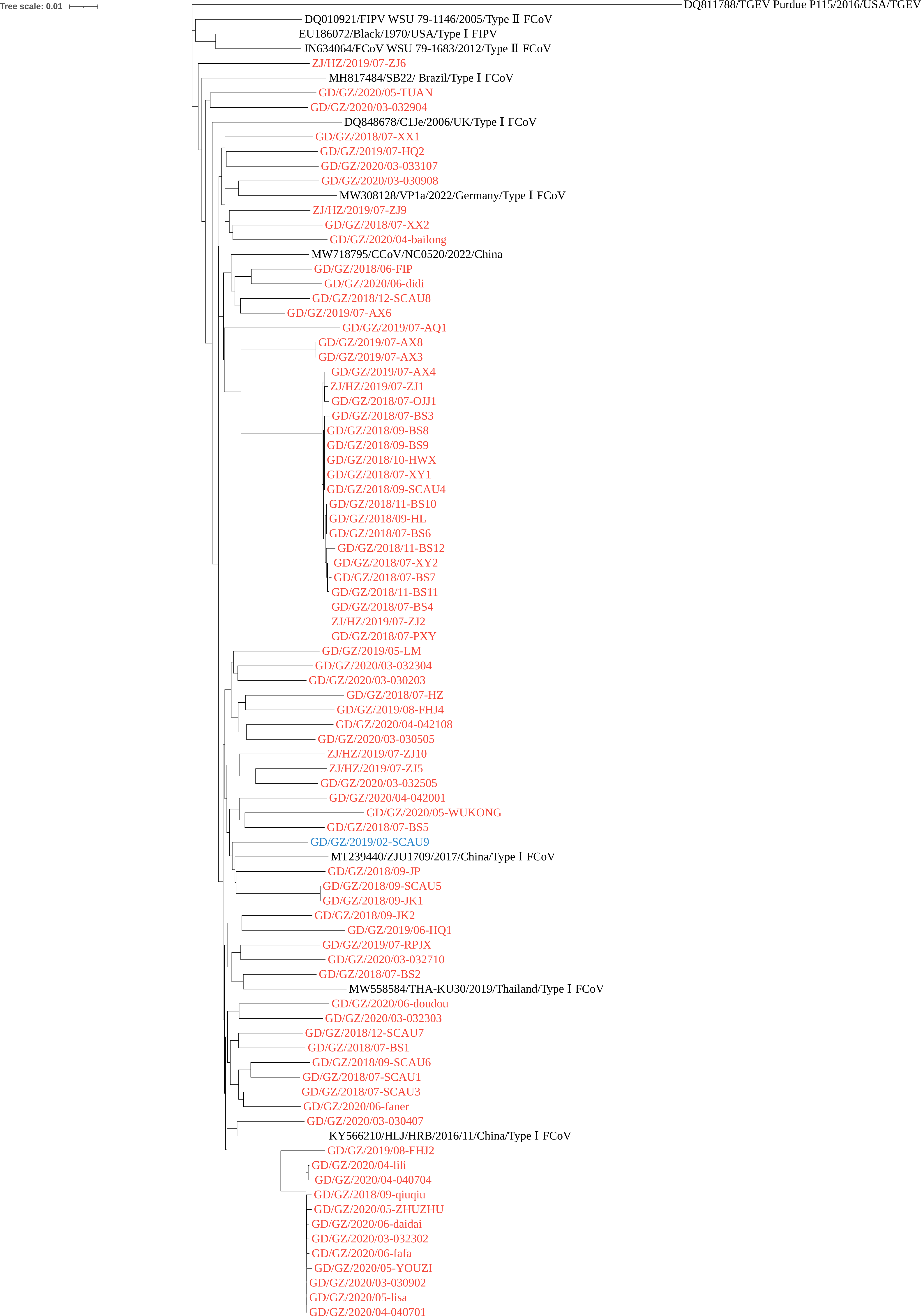
**FIGURE S2** Genetic evolutionary tree of FIPV N genes. The strains identified as type I FIPV are shown in red, and the blue represents the type II FIPV. Black represents the reference strains of FCoV downloaded from GenBank. Neighbor-joining trees were constructed with 1,000 bootstrap replicates.

Figure S3 The nucleotide homology of S genes of FIPV type I strains with reference strains. A and B are the nucleotide homology of S genes obtained in this study with the reference strains (Black, No. EU186072; SB22, No. MH817484; HRB201611, No. KY566210; UCD1,No. AB088222).


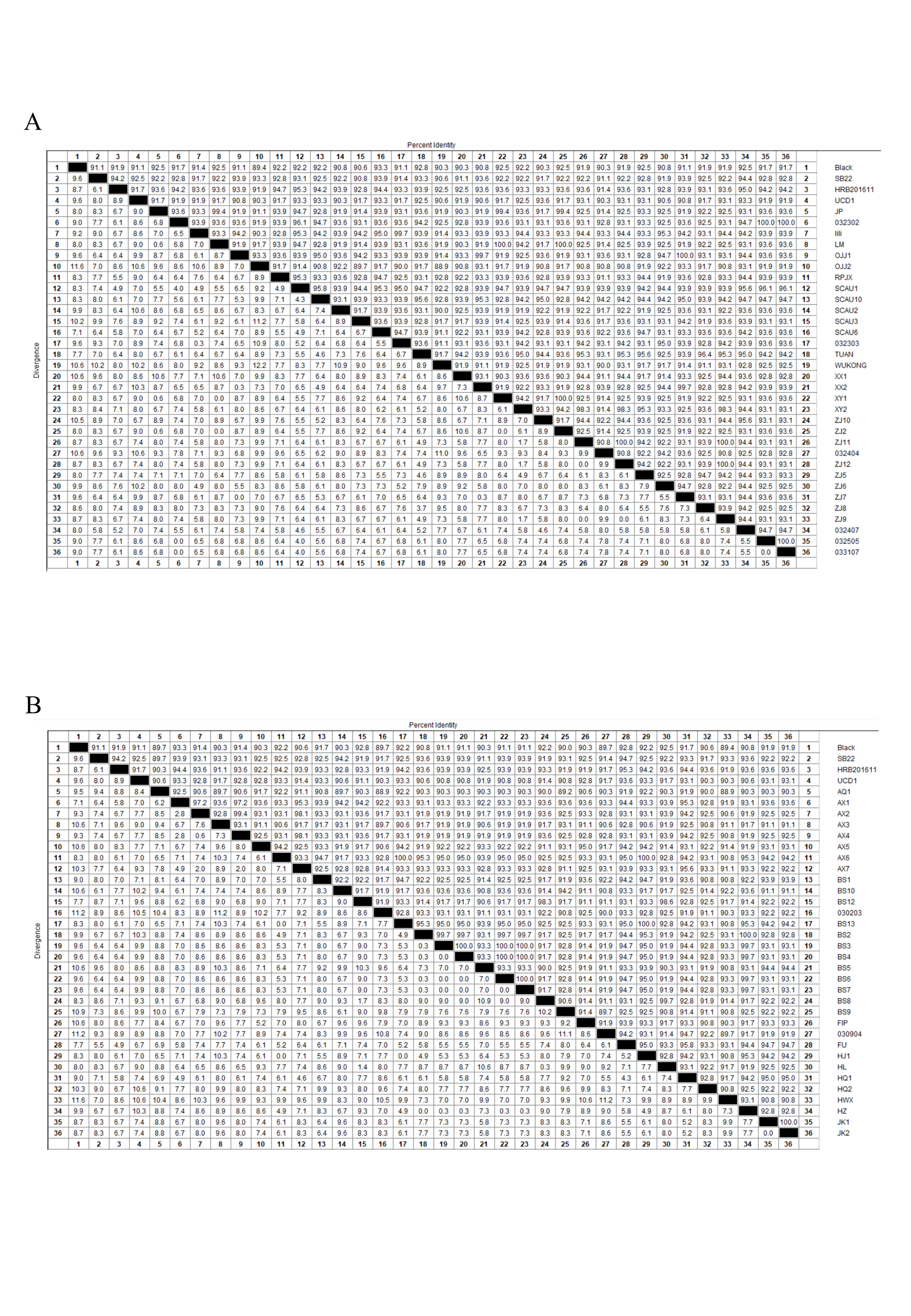


Figure S4 The nucleotide and amino acid homology of FIPV N genes. A is the amino acid homology of the obtained FIPV N genes between the reference strains (HRB201611, No. KY566210; ZJU1709, No. MT239440; FIPV 79-1146, No. DQ010921; WSU 79-1683, No. JN634064; Black, No. EU186072); B is the nucleotide homology of the obtained FIPV N genes between the reference strains (HRB201611, No. KY566210; ZJU1709, No. MT239440; FIPV 79-1146, No. DQ010921; WSU 79-1683, No. JN634064; Black, No. EU186072).
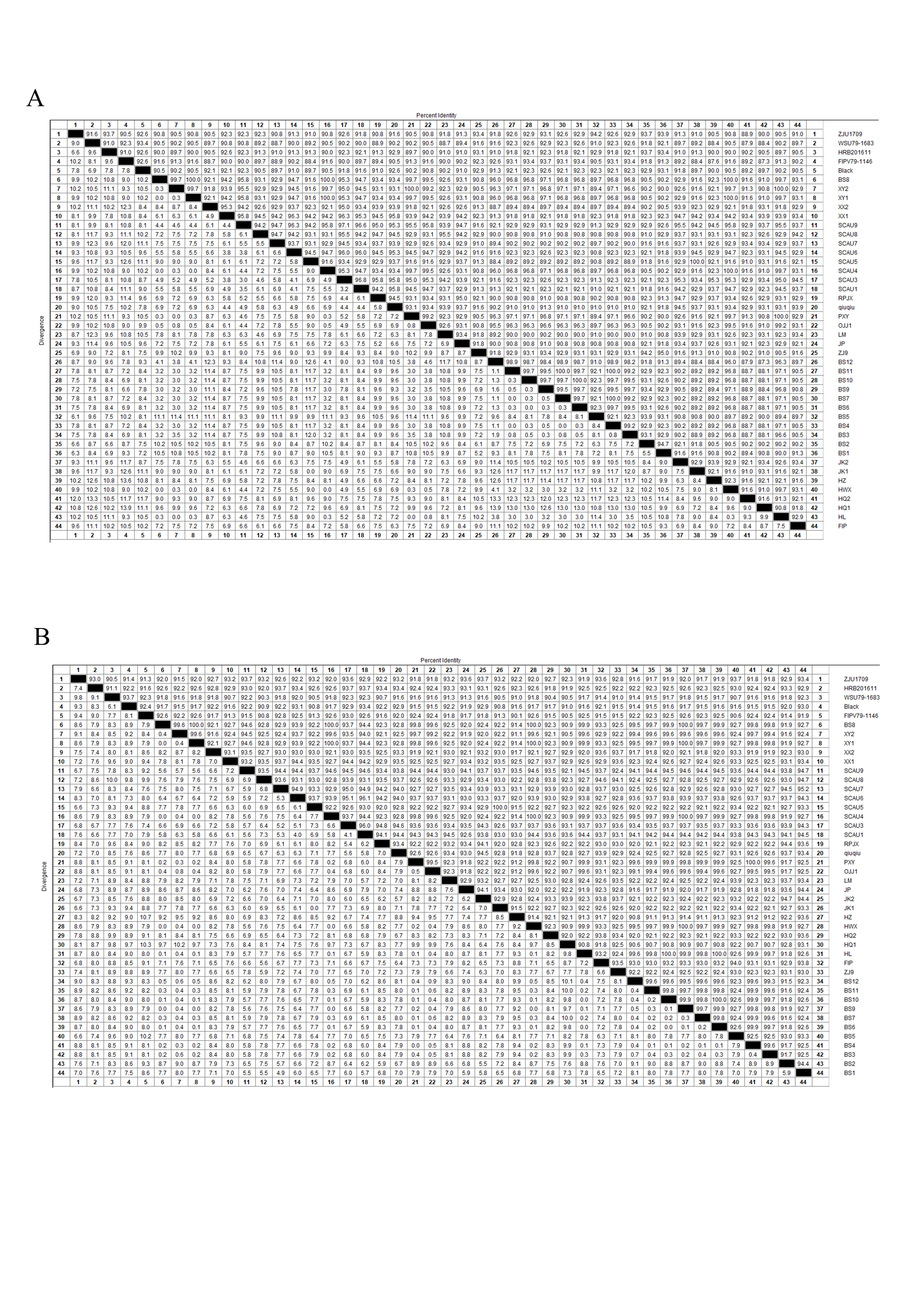


**Figure S5** Screening of antibodies by IFA. HEK 293T cells were transfected with pCMV-MYC-BS8-N plasmid, and the supernatant of each fusion-positive cell line was used as the primary antibody for IFA.


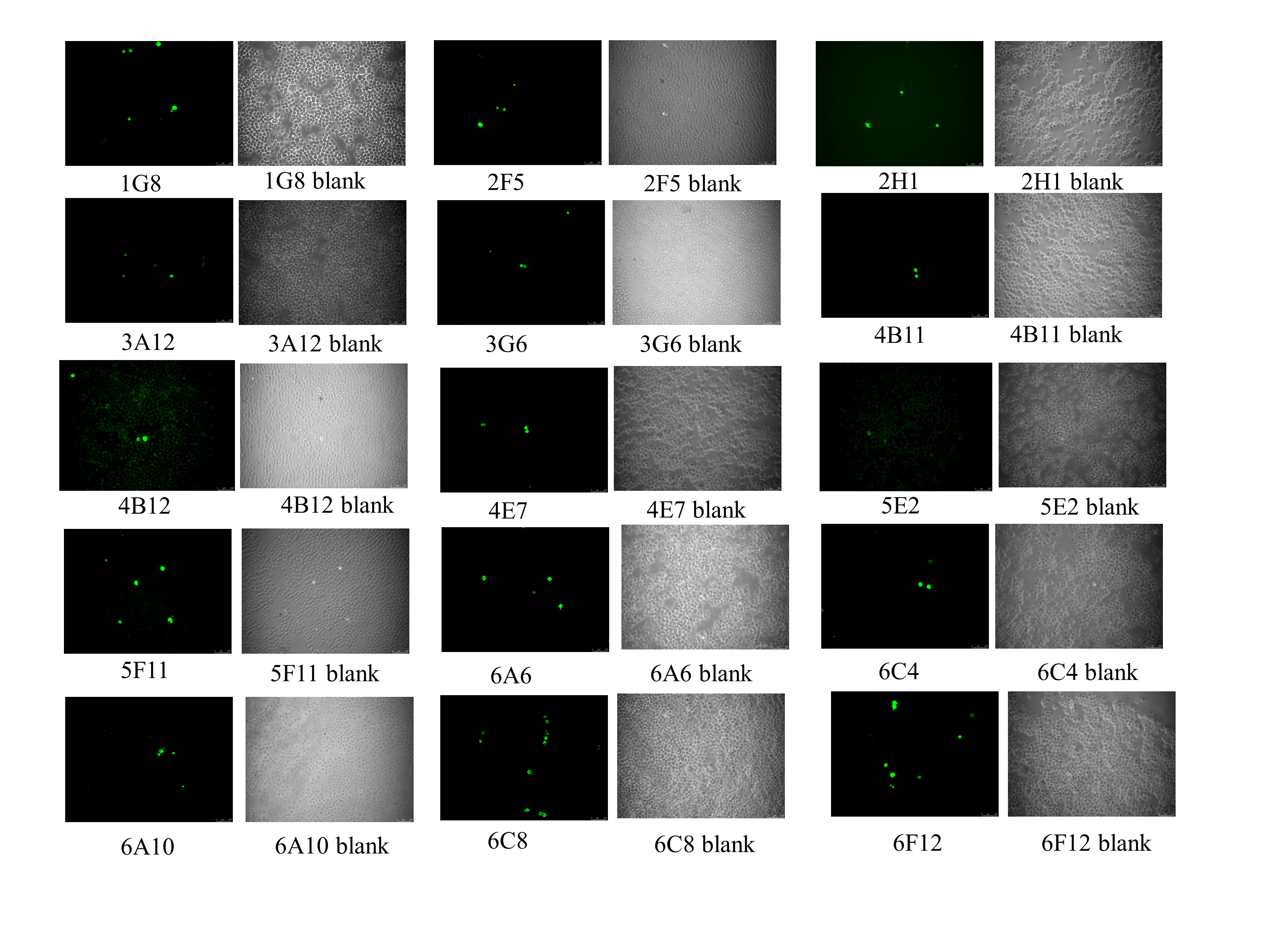


**
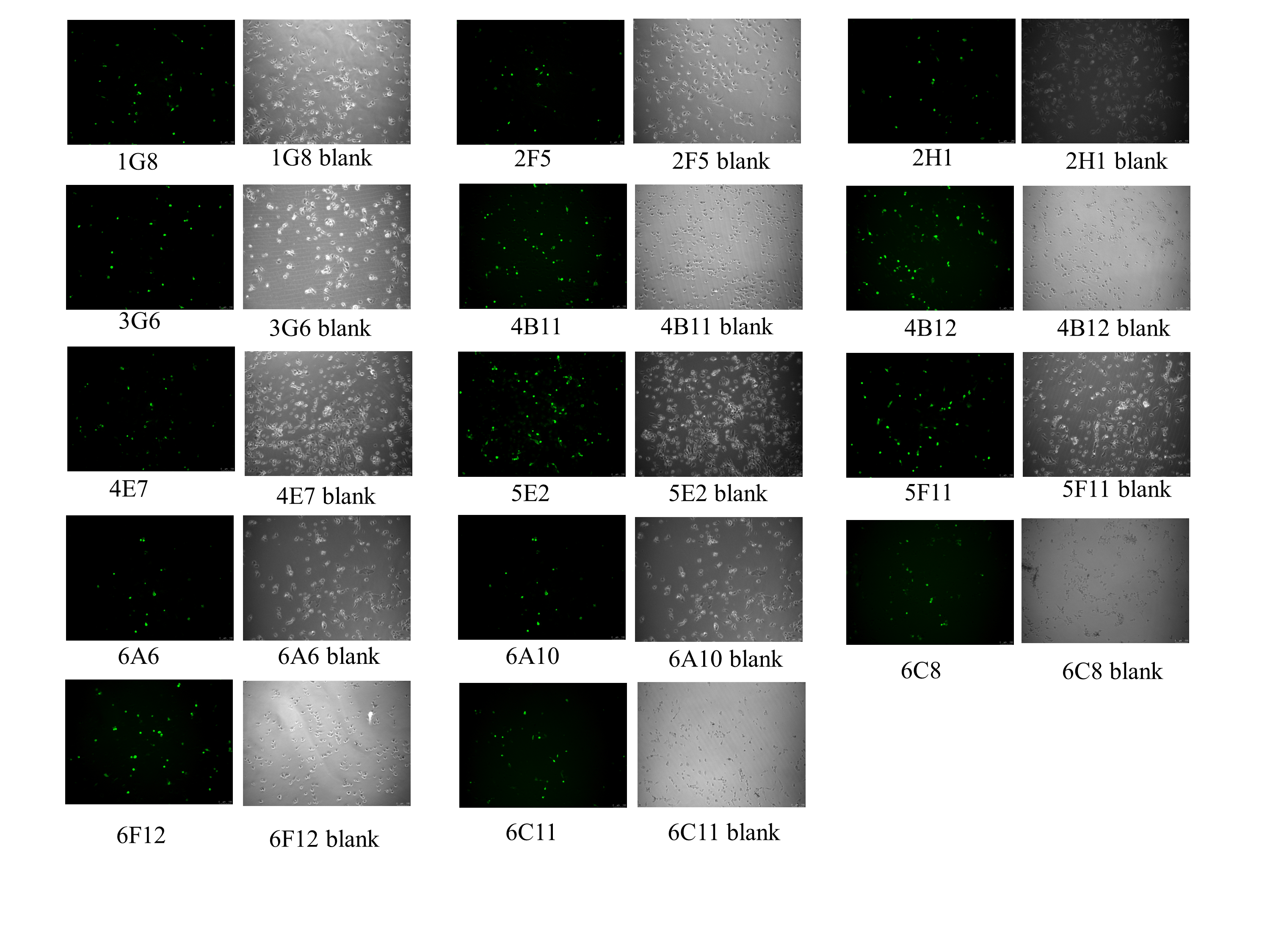
FIGURE S6** Screening of antibodies by IFA. Fcwf-4 cells were transfected with pCMV-MYC-BS8-N plasmid, and the supernatant of each fusion-positive cell line was used as the primary antibody for IFA.

**
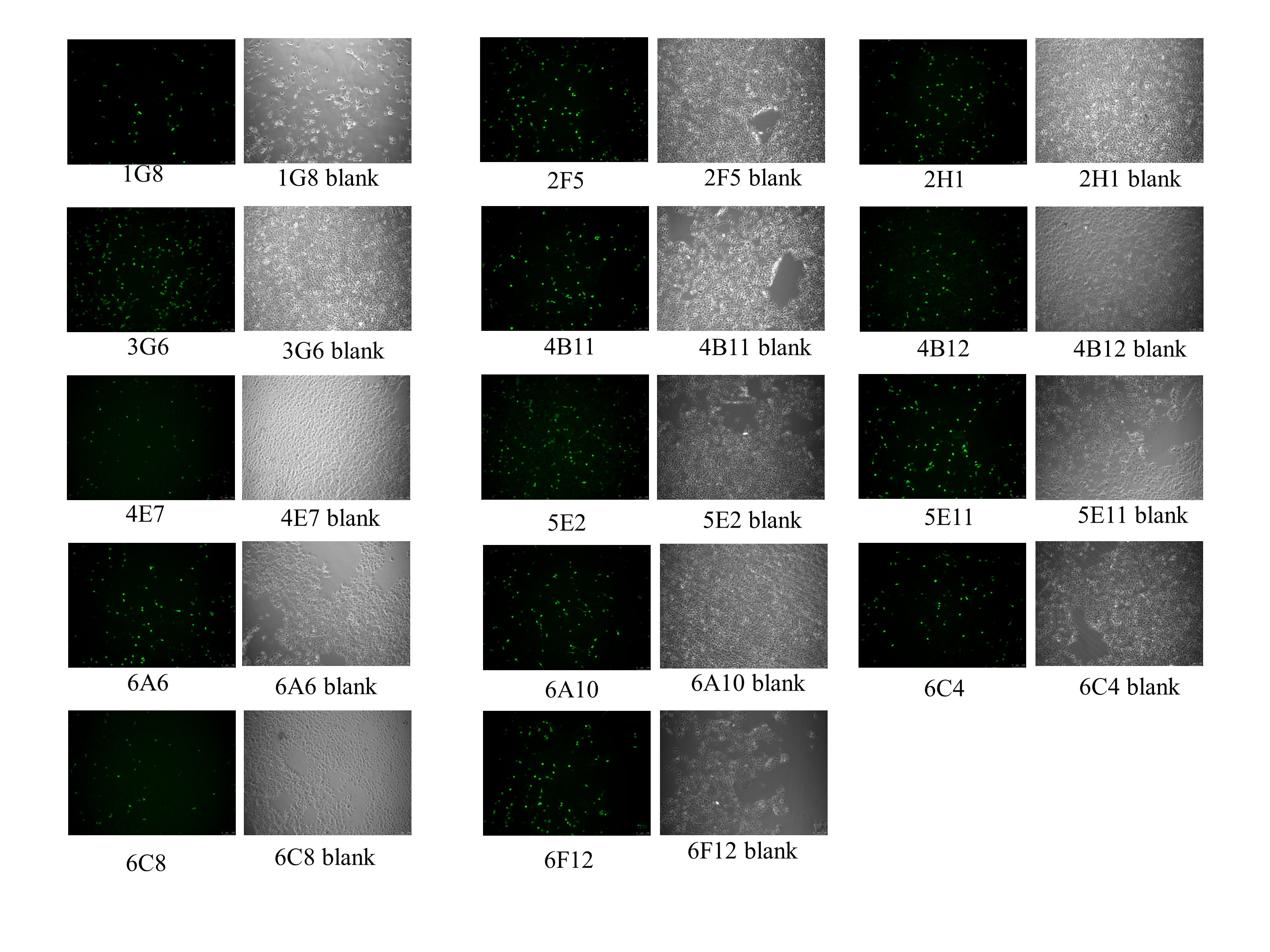
FIGURE S7** Screening of antibodies by IFA. CrFK cells were transfected with pCMV-MYC-BS8-N plasmid, and the supernatant of each fusion-positive cell line was used as the primary antibody for IFA.
