# Supplementary material for: A Combined Method Based on the FIPV N Monoclonal Antibody Immunofluorescence Assay and RT-nPCR Method for the Rapid Diagnosis of FIP-Suspected Ascites
Source: Transbound Emerg Dis. 2023 Mar 28;2023:8429106. doi: 10.1155/2023/8429106 (PMC12017028; doi:10.1155/2023/8429106)
Supplement: Supplementary Materials — Supplemental Figure S1: electrophoresis results of FIPV 3UTR, N, and S genes in some samples. Supplemental Figure S2: genetic evolutionary tree of FIPV N genes. Supplemental Figure S3: the nucleotide homology of S genes of FIPV type I strains with reference strains. Supplemental Figure S4: the nucleotide and amino acid homology of FIPV N genes. Supplemental Figure S5–S7: screening of antibodies by IFA. Supplemental Table S1: the information of the samples obtained sequences of N and S gene of FIPV. [file 8429106.f1.zip › Updated Supplemental Table (1).docx]

Table S1 The information of the samples obtained sequences of N and S gene of FIPV

| NO. | Strain name | Collection date | Geographical location | Gender | Sample source | Detection of FIPV based on N gene | Detection of FIPV based on S gene | Genotying of FIPV based on partial S gene | | |
| --- | --- | --- | --- | --- | --- | --- | --- | --- | --- | --- |
|  |  |  |  |  |  |  |  | Type Ⅰ FIPV | | Type Ⅱ FIPV |
| 1 | GD/GZ/2018/06/FIP | Jun-2018 | Guangzhou city, Guangdong | NA | AF | + | + | + | | - |
| 2 | GD/GZ/2018/07/BS1 | Jul-2018 | Guangzhou city, Guangdong | M | AF | + | + | + | | - |
| 3 | GD/GZ/2018/07/BS2 | Jul-2018 | Guangzhou city, Guangdong | NA | AF | + | + | + | | - |
| 4 | GD/GZ/2018/07/BS3 | Jul-2018 | Guangzhou city, Guangdong | M | AF | + | + | + | | - |
| 5 | GD/GZ/2018/07/BS4 | Jul-2018 | Guangzhou city, Guangdong | F | AF | + | + | + | | - |
| 6 | GD/GZ/2018/07/BS5 | Jul-2018 | Guangzhou city, Guangdong | F | AF | + | + | + | | - |
| 7 | GD/GZ/2018/07/BS6 | Jul-2018 | Guangzhou city, Guangdong | F | AF | + | + | + | | - |
| 8 | GD/GZ/2018/07/BS7 | Jul-2018 | Guangzhou city, Guangdong | M | AF | + | + | + | | - |
| 9 | GD/GZ/2018/07/HZ | Jul-2018 | Guangzhou city, Guangdong | M | AF | + | + | + | | - |
| 10 | GD/GZ/2018/07/OJJ1 | Jul-2018 | Guangzhou city, Guangdong | M | AF | + | + | + | | - |
| 11 | GD/GZ/2018/07/OJJ2 | Jul-2018 | Guangzhou city, Guangdong | M | AF | - | + | + | | - |
| 12 | GD/GZ/2018/07/SCAU1 | Jul-2018 | Guangzhou city, Guangdong | M | AF | + | + | + | | - |
| 13 | GD/GZ/2018/07/SCAU3 | Jul-2018 | Guangzhou city, Guangdong | F | AF | + | + | + | | - |
| 14 | GD/GZ/2018/07/XX1 | Jul-2018 | Guangzhou city, Guangdong | F | AF | + | + | + | | - |
| 15 | GD/GZ/2018/07/XX2 | Jul-2018 | Guangzhou city, Guangdong | F | AF | + | + | + | | - |
| 16 | GD/GZ/2018/07/XY2 | Jul-2018 | Guangzhou city, Guangdong | M | AF | + | + | + | | - |
| 17 | GD/GZ/2018/07-PXY | Jul-2018 | Guangzhou city, Guangdong | M | AF | + | - |  | |  |
| 18 | GD/GZ/2018/09/BS8 | Sep-2018 | Guangzhou city, Guangdong | F | AF | + | + | + | | - |
| 19 | GD/GZ/2018/09/BS9 | Sep-2018 | Guangzhou city, Guangdong | M | AF | + | + | + | | - |
| NO. | Strain name | Collection date | Geographical location | Gender | Sample source | Detection of FIPV based on N gene | Detection of FIPV based on S gene | Genotying of FIPV based on partial S gene | | |
|  |  |  |  |  |  |  |  | Type Ⅰ FIPV | | Type Ⅱ FIPV |
| 20 | GD/GZ/2018/09/HL | Sep-2018 | Guangzhou city, Guangdong | M | AF | + | + | + | | - |
| 21 | GD/GZ/2018/09/JK1 | Sep-2018 | Guangzhou city, Guangdong | M | AF | + | + | + | | - |
| 22 | GD/GZ/2018/09/JK2 | Sep-2018 | Guangzhou city, Guangdong | F | AF | + | + | + | | - |
| 23 | GD/GZ/2018/09/JP | Sep-2018 | Guangzhou city, Guangdong | F | AF | + | + | + | | - |
| 24 | GD/GZ/2018/09/SCAU6 | Sep-2018 | Guangzhou city, Guangdong | M | AF | + | + | + | | - |
| 25 | GD/GZ/2018/09-qiuqiu | Sep-2018 | Guangzhou city, Guangdong | F | FA | + | - |  | |  |
| 26 | GD/GZ/2018/09-SCAU4 | Sep-2018 | Guangzhou city, Guangdong | F | AF | + | - |  | |  |
| 27 | GD/GZ/2018/09-SCAU5 | Sep-2018 | Guangzhou city, Guangdong | M | AF | + | - |  | |  |
| 28 | GD/GZ/2018/10/HJ1 | Oct-2018 | Guangzhou city, Guangdong | M | AF | - | + | + | | - |
| 29 | GD/GZ/2018/10/HWX | Oct-2018 | Guangzhou city, Guangdong | M | AF | + | + | + | | - |
| 30 | GD/GZ/2018/11/BS10 | Nov-2018 | Guangzhou city, Guangdong | F | AF | + | + | + | | - |
| 31 | GD/GZ/2018/11/BS12 | Nov-2018 | Guangzhou city, Guangdong | F | AF | + | + | + | | - |
| 32 | GD/GZ/2018/11/BS13 | Nov-2018 | Guangzhou city, Guangdong | F | AF | - | + |  | | - |
| 33 | GD/GZ/2018/11/BS13 | Nov-2018 | Guangzhou city, Guangdong | N | AF | - | + | + | | - |
| 34 | GD/GZ/2018/11-BS11 | Nov-2018 | Guangzhou city, Guangdong | F | AF | + | - |  | |  |
| 35 | GD/GZ/2018/12-SCAU7 | Dec-2018 | Guangzhou city, Guangdong | M | AF | + | - |  | |  |
| 36 | GD/GZ/2018/12-SCAU8 | Dec-2018 | Guangzhou city, Guangdong | M | AF | + | - |  | |  |
| 37 | GD/GZ/2019/02/SCAU9 | Feb-2019 | Guangzhou city, Guangdong | M | AF | + | + | - | | + |
| 38 | GD/GZ/2019/05/LM | May-2019 | Guangzhou city, Guangdong | M | AF | + | + | + | | - |
| 39 | GD/GZ/2019/06/HQ1 | Jun-2019 | Guangzhou city, Guangdong | M | AF | + | + | + | | - |
| 40 | GD/GZ/2018/07/SCAU2 | Jul-2019 | Guangzhou city, Guangdong | M | AF | - | + | + | | - |
| NO. | Strain name | Collection date | Geographical location | Gender | Sample source | Detection of FIPV based on N gene | Detection of FIPV based on S gene | Genotying of FIPV based on partial S gene | | |
|  |  |  |  |  |  |  |  | Type Ⅰ FIPV | | Type Ⅱ FIPV |
| 41 | GD/GZ/2018/07/XY1 | Jul-2019 | Guangzhou city, Guangdong | M | AF | + | + | + | | - |
| 42 | GD/GZ/2019/07/AQ1 | Jul-2019 | Guangzhou city, Guangdong | NA | AF | + | + | + | | - |
| 43 | GD/GZ/2019/07/HQ2 | Jul-2019 | Guangzhou city, Guangdong | NA | AF | + | + | + | | - |
| 44 | GD/GZ/2019/07/RPJX | Jul-2019 | Guangzhou city, Guangdong | NA | AF | + | + | + | | - |
| 45 | GD/GZ/2019/07-AX8 | Jul-2019 | Guangzhou city, Guangdong | M | AF | + | - |  | |  |
| 46 | ZJ/HZ/2019/07/ZJ10 | Jul-2019 | Hangzhou city, Zhejiang | M | AF | + | + | + | | - |
| 47 | ZJ/HZ/2019/07/ZJ11 | Jul-2019 | Hangzhou city, Zhejiang | M | AF | + | + | + | | - |
| 48 | ZJ/HZ/2019/07/ZJ12 | Jul-2019 | Hangzhou city, Zhejiang | M | AF | - | + | + | | - |
| 49 | ZJ/HZ/2019/07/ZJ2 | Jul-2019 | Hangzhou city, Zhejiang | M | AF | + | + | + | | - |
| 50 | ZJ/HZ/2019/07-ZJ1 | Jul-2019 | Hangzhou city, Zhejiang | M | AF | + | - |  | |  |
| 51 | GD/GZ/2019/08/AX1 | Aug-2019 | Guangzhou city, Guangdong | F | AF | - | + | + | | - |
| 52 | GD/GZ/2019/08/AX2 | Aug-2019 | Guangzhou city, Guangdong | M | AF | + | + | + | | - |
| 53 | GD/GZ/2019/08/AX3 | Aug-2019 | Guangzhou city, Guangdong | F | AF | + | + | + | | - |
| 54 | GD/GZ/2019/08/AX4 | Aug-2019 | Guangzhou city, Guangdong | M | AF | + | + | + | | - |
| 55 | GD/GZ/2019/08/AX5 | Aug-2019 | Guangzhou city, Guangdong | F | AF | - | + | + | | - |
| 56 | GD/GZ/2019/08/AX6 | Aug-2019 | Guangzhou city, Guangdong | M | AF | + | + | + | | - |
| 57 | GD/GZ/2019/08/AX7 | Aug-2019 | Guangzhou city, Guangdong | F | AF | - | + | + | | - |
| 58 | GD/GZ/2019/08-FHJ2 | Aug-2019 | Guangzhou city, Guangdong | NA | AF | + | - |  | |  |
| 59 | GD/GZ/2019/08-FHJ4 | Aug-2019 | Guangzhou city, Guangdong | M | AF | + | - |  | |  |
| 60 | GD/GZ/2019/09/SCAU10 | Sep-2019 | Guangzhou city, Guangdong | M | AF | - | + | + | | - |
| 61 | GD/GZ/2020/03/030203 | Mar-2020 | Guangzhou city, Guangdong | M | AF | + | + | + | | - |
| NO. | Strain name | Collection date | Geographical location | Gender | Sample source | Detection of FIPV based on N gene | Detection of FIPV based on S gene | Genotying of FIPV based on partial S gene | | |
|  |  |  |  |  |  |  |  | Type Ⅰ FIPV | | Type Ⅱ FIPV |
| 62 | GD/GZ/2020/03/030407 | Mar-2020 | Guangzhou city, Guangdong | F | AF | + | - |  | |  |
| 63 | GD/GZ/2020/03/030505 | Mar-2020 | Guangzhou city, Guangdong | F | AF | + | - |  | |  |
| 64 | GD/GZ/2020/03/030902 | Mar-2020 | Guangzhou city, Guangdong | M | AF | + | - |  | |  |
| 65 | GD/GZ/2020/03/030904 | Mar-2020 | Guangzhou city, Guangdong | NA | AF | - | + | + | | - |
| 66 | GD/GZ/2020/03/030908 | Mar-2020 | Guangzhou city, Guangdong | M | AF | + | - |  | |  |
| 67 | GD/GZ/2020/03/032302 | Mar-2020 | Guangzhou city, Guangdong | M | AF | + | + | + | | - |
| 68 | GD/GZ/2020/03/032303 | Mar-2020 | Guangzhou city, Guangdong | NA | AF | - | + | + | | - |
| 69 | GD/GZ/2020/03/032304 | Mar-2020 | Guangzhou city, Guangdong | M | AF | + | - |  | |  |
| 70 | GD/GZ/2020/03/032404 | Mar-2020 | Guangzhou city, Guangdong | NA | AF | - | + | + | | - |
| 71 | GD/GZ/2020/03/032407 | Mar-2020 | Guangzhou city, Guangdong | F | AF | - | + | + | | - |
| 72 | GD/GZ/2020/03/032505 | Mar-2020 | Guangzhou city, Guangdong | M | AF | + | + | + | | - |
| 73 | GD/GZ/2020/03/033107 | Mar-2020 | Guangzhou city, Guangdong | F | AF | + | + | + | | - |
| 74 | GD/GZ/2020/03-032710 | Mar-2020 | Guangzhou city, Guangdong | NA | AF | + | - |  | |  |
| 75 | GD/GZ/2020/03-032904 | Mar-2020 | Guangzhou city, Guangdong | F | AF | + | - |  | |  |
| 75 | GD/GZ/2020/05-YOUZI | Mar-2020 | Guangzhou city, Guangdong | F | AF | + | - |  | |  |
| 77 | GD/GZ/2020/05-ZHUZHU | Mar-2020 | Guangzhou city, Guangdong | NA | AF | + | - |  | |  |
| 78 | ZJ/HZ/2020/03/ZJ5 | Mar-2020 | Hangzhou city, Zhejiang | NA | AF | - | + | + | | - |
| 79 | ZJ/HZ/2020/03/ZJ6 | Mar-2020 | Hangzhou city, Zhejiang | F | AF | + | + | + | | - |
| 80 | ZJ/HZ/2020/03/ZJ7 | Mar-2020 | Hangzhou city, Zhejiang | M | AF | - | + | + | |  |
| 81 | ZJ/HZ/2020/03/ZJ8 | Mar-2020 | Hangzhou city, Zhejiang | F | AF | - | + | + | | - |
| 82 | ZJ/HZ/2020/03/ZJ9 | Mar-2020 | Hangzhou city, Zhejiang | F | AF | + | + | + | | - |
| NO. | Strain name | Collection date | Geographical location | Gender | Sample source | Detection of FIPV based on N gene | Detection of FIPV based on S gene | Genotying of FIPV based on partial S gene | | |
|  |  |  |  |  |  |  |  | Type Ⅰ FIPV | Type Ⅱ FIPV | |
| 83 | GD/GZ/2020/04/FU | Apr-2020 | Guangzhou city, Guangdong | NA | AF | - | + | + | | - |
| 84 | GD/GZ/2020/04/lili | Apr-2020 | Guangzhou city, Guangdong | M | AF | + | + | + | | - |
| 85 | GD/GZ/2020/04-040701 | Apr-2020 | Guangzhou city, Guangdong | F | AF | + | - |  | |  |
| 86 | GD/GZ/2020/04-040704 | Apr-2020 | Guangzhou city, Guangdong | F | AF | + | - |  | |  |
| 87 | GD/GZ/2020/04-042001 | Apr-2020 | Guangzhou city, Guangdong | F | AF | + | - |  | |  |
| 88 | GD/GZ/2020/04-042108 | Apr-2020 | Guangzhou city, Guangdong | M | AF | + | - |  | |  |
| 89 | GD/GZ/2020/04-bailong | Apr-2020 | Guangzhou city, Guangdong | M | AF | + | - |  | |  |
| 90 | GD/GZ/2020/05/TUAN | May-2020 | Guangzhou city, Guangdong | M | AF | + | + | + | | - |
| 91 | GD/GZ/2020/05/WUKONG | May-2020 | Guangzhou city, Guangdong | M | AF | + | + | + | | - |
| 92 | GD/GZ/2020/05-lisa | May-2020 | Guangzhou city, Guangdong | F | AF | + | - |  | |  |
| 93 | GD/GZ/2020/06-daidai | Jun-2020 | Guangzhou city, Guangdong | M | AF | + | - |  | |  |
| 94 | GD/GZ/2020/06-doudou | Jun-2020 | Guangzhou city, Guangdong | F | AF | + | - |  | |  |
| 95 | GD/GZ/2020/06-fafa | Jun-2020 | Guangzhou city, Guangdong | M | AF | + | - |  | |  |
| 96 | GD/GZ/2020/06-faner | Jun-2020 | Guangzhou city, Guangdong | F | AF | + | - |  | |  |

Note: For gender, F=female, and M=male, NA=not available, AF=ascitic fluid. “+” represents positive results. “-” represents negative results.
